# Clear Cell Myoepithelial Carcinoma of the Clavicle: A Case Report

**DOI:** 10.7759/cureus.99517

**Published:** 2025-12-18

**Authors:** Iosafat Pinto, Panagiotis Konstantinou, Evangelos Petsatodis, Tryfon Ditsios, Konstantinos Ditsios

**Affiliations:** 1 Orthopedics and Trauma, General Hospital of Imathia, Veria Health Unit, Veria, GRC; 2 2nd Orthopedic Department, Aristotle University Thessaloniki, Thessaloniki, GRC; 3 Department of Interventional Radiology, G Papanikolaou General Hospital, Thessaloniki, GRC; 4 Megisti Regional Medical Center, Andreas G Papandreou General Hospital of Rhodes, Kastellorizo, GRC; 5 2nd Orthopedic Department, Aristotle University of Thessaloniki, Thessaloniki, GRC

**Keywords:** bone myoepithelial carcinoma, clavicle tumor, clear cell tumor, fibular autograft reconstruction, myoepithelioma

## Abstract

Clear cell myoepithelial carcinoma involving the clavicle is an exceptionally rare malignancy. Its nonspecific clinical and radiologic features pose significant diagnostic challenges, often mimicking more common primary bone tumors such as chondrosarcoma and osteosarcoma. We report the case of a 53-year-old male with a slowly enlarging, painless supraclavicular mass present for nearly a decade. Imaging studies suggested chondrosarcoma, and an initial fine-needle biopsy yielded a diagnosis of grade II chondrosarcoma. Staging showed no metastatic disease, and the patient underwent en bloc resection of the tumor with the affected clavicular segment, followed by reconstruction using a fibular autograft fixed with dual plating. Final histopathology and a comprehensive immunohistochemical panel confirmed the diagnosis of clear cell myoepithelial carcinoma. All margins were negative, and postoperative recovery was uneventful. This case highlights the diagnostic complexity of rare clavicular tumors and underscores the importance of thorough histopathologic evaluation, as imaging and needle biopsy may occasionally provide misleading diagnoses in uncommon bone lesions. Wide surgical excision remains the cornerstone of treatment, and reconstruction should be individualized based on defect size and regional anatomical considerations. Continued reporting of such rare cases is essential to improve diagnostic accuracy and guide treatment strategies

## Introduction

Myoepithelial carcinomas (MC) are uncommon neoplasms that arise primarily in the parotid gland, submandibular gland, and palate [1]. Primary osseous cases are exceedingly rare, with only isolated reports involving long bones or the axial skeleton [2]. Although bone myoepithelial carcinomas are usually intraosseous tumors, juxtacortical lesions also have been reported [3]. There are a diverse number of tumor cell types seen in this neoplasm, and most cases demonstrate multiple cell types such as epithelioid, clear cell, plasmacytoid, spindled, and mixed [1]. Clear cell tumor cells with myoepithelial features are a rare subtype of myoepithelial carcinoma. These neoplasms are infiltrative and usually cause local destruction to nearby tissues. Metastasis is uncommon but has been seen in late stages of disease [4].

Due to the rarity of bone myoepithelial carcinoma, standardized treatment guidelines are lacking. Current evidence supports wide surgical excision of the primary tumor as the cornerstone of management [5]. If metastasis in the adjacent lymph nodes is not detected, dissection of the lymph nodes is unnecessary [6,7]. Adjuvant radiotherapy may be considered for close margins or recurrent disease, although its role remains undefined [8]. In addition, postsurgical chemotherapy is administered to treat systemic metastasis, similar to other cancers. We present a unique case of clear cell myoepithelial carcinoma (CCMC) of the clavicle, highlighting diagnostic challenges and surgical treatment considerations in this exceptionally uncommon neoplasm.

## Case presentation

**Figure 1:**
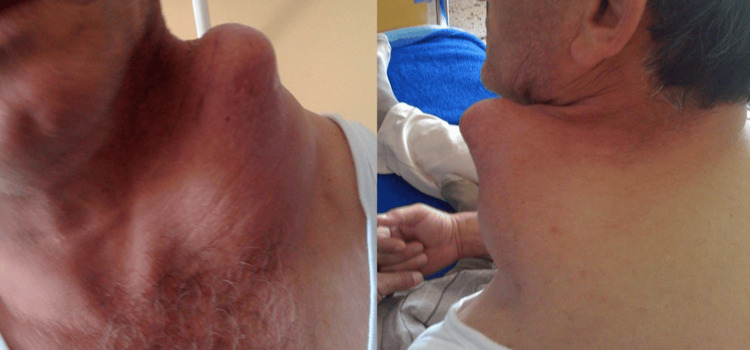
Clinical presentation of a large, subcutaneous left supraclavicular mass on the medial side of the clavicle

A 53-year-old male presented with a large, painless, left supraclavicular mass that he reported had first appeared approximately ten years earlier and had slowly increased in size (Figure 1). He had not previously sought medical evaluation due to an intense fear of hospitals and tumors. His medical history was unremarkable, and physical examination demonstrated a firm, non-tender mass without overlying skin changes. Neurovascular examination of the left upper limb was normal, and routine laboratory tests were within normal limits. 

**Figure 2:**
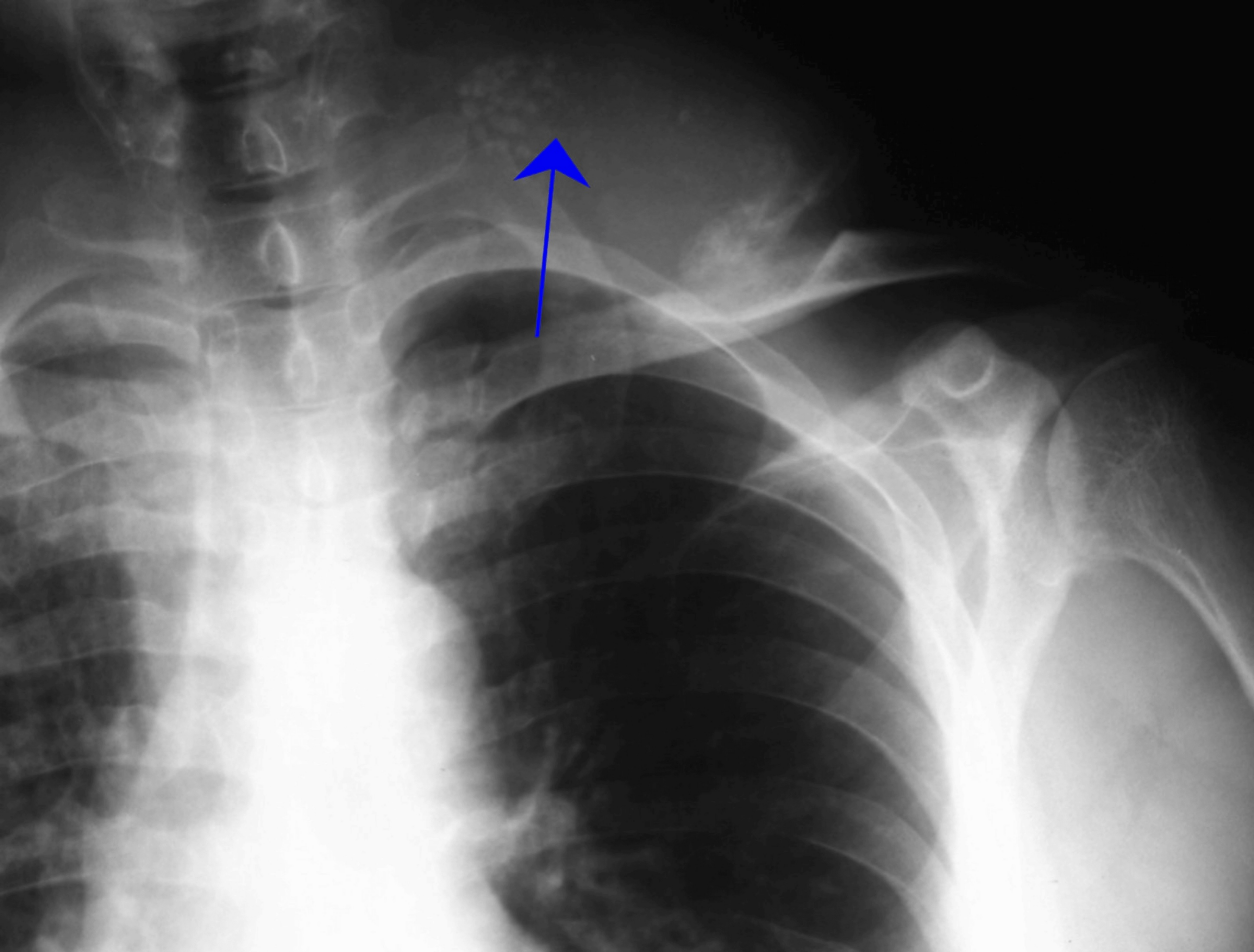
X-ray of the left clavicle shows a lesion on the medial and superior aspect of the clavicle

**Figure 3:**
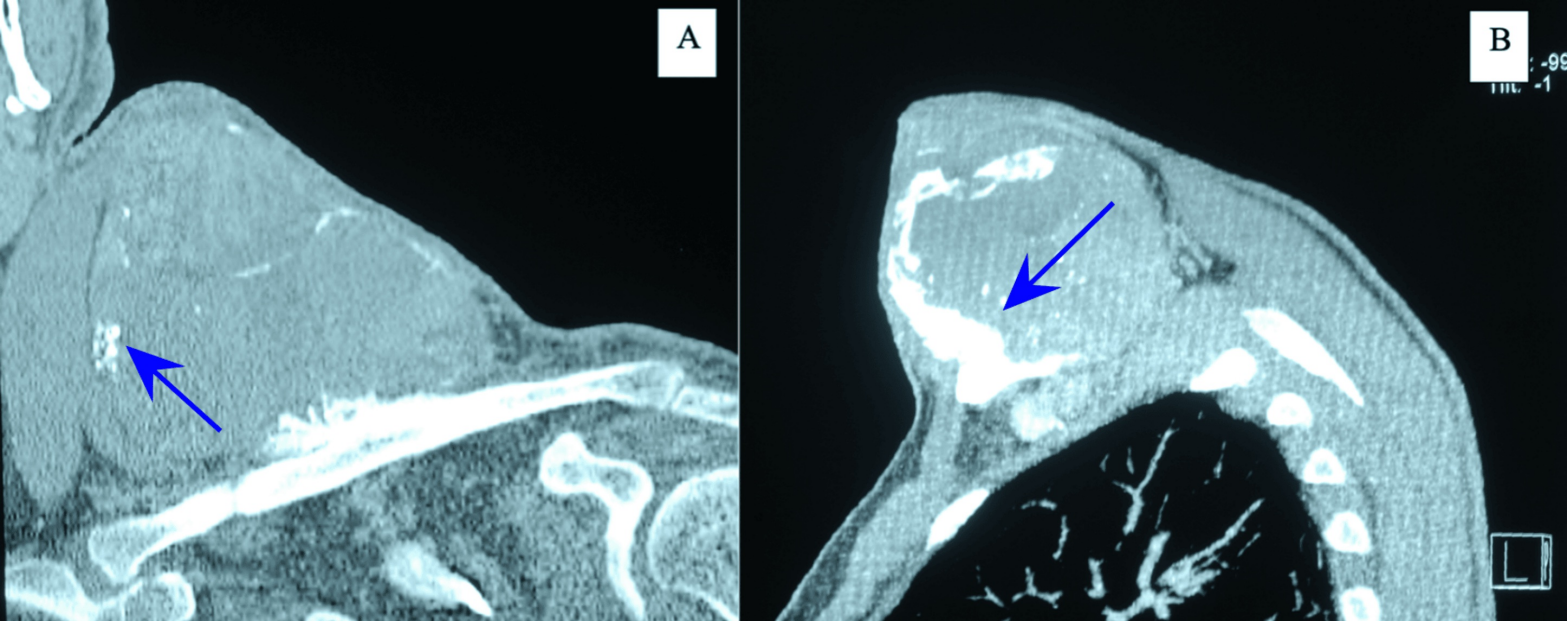
CT scan of the tumor CT scan of the tumor shows a juxtacortical, well-defined, lobular lesion with invasion of the surrounding soft tissues and mineralization along its margins. A: coronal view; B: sagittal view

**Figure 4:**
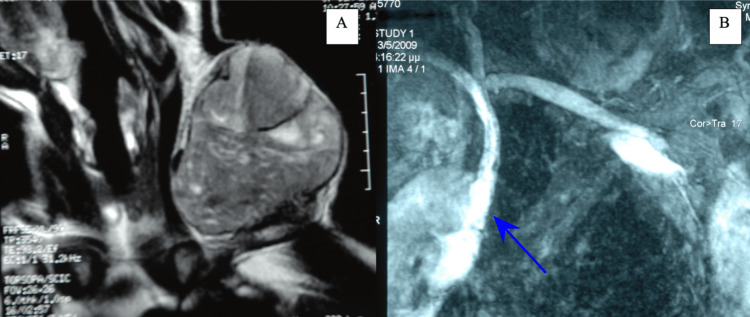
MRI and MRA images of the mass A: MRI shows a well-defined, multi-lobulated mass with heterogeneous signal intensity due to tissue mineralization. B: MRA coronal view shows peripheral/septal enhancement.

X-rays revealed a well-defined juxtacortical clavicular mass with lobulated contours and internal calcified foci, associated with pressure remodeling and thinning of the underlying cortex, prompting further evaluation with CT and MRI, where the chondroid-type calcifications and lobulated morphology initially favored a diagnosis of chondrosarcoma (Figures 2-4).

**Figure 5:**
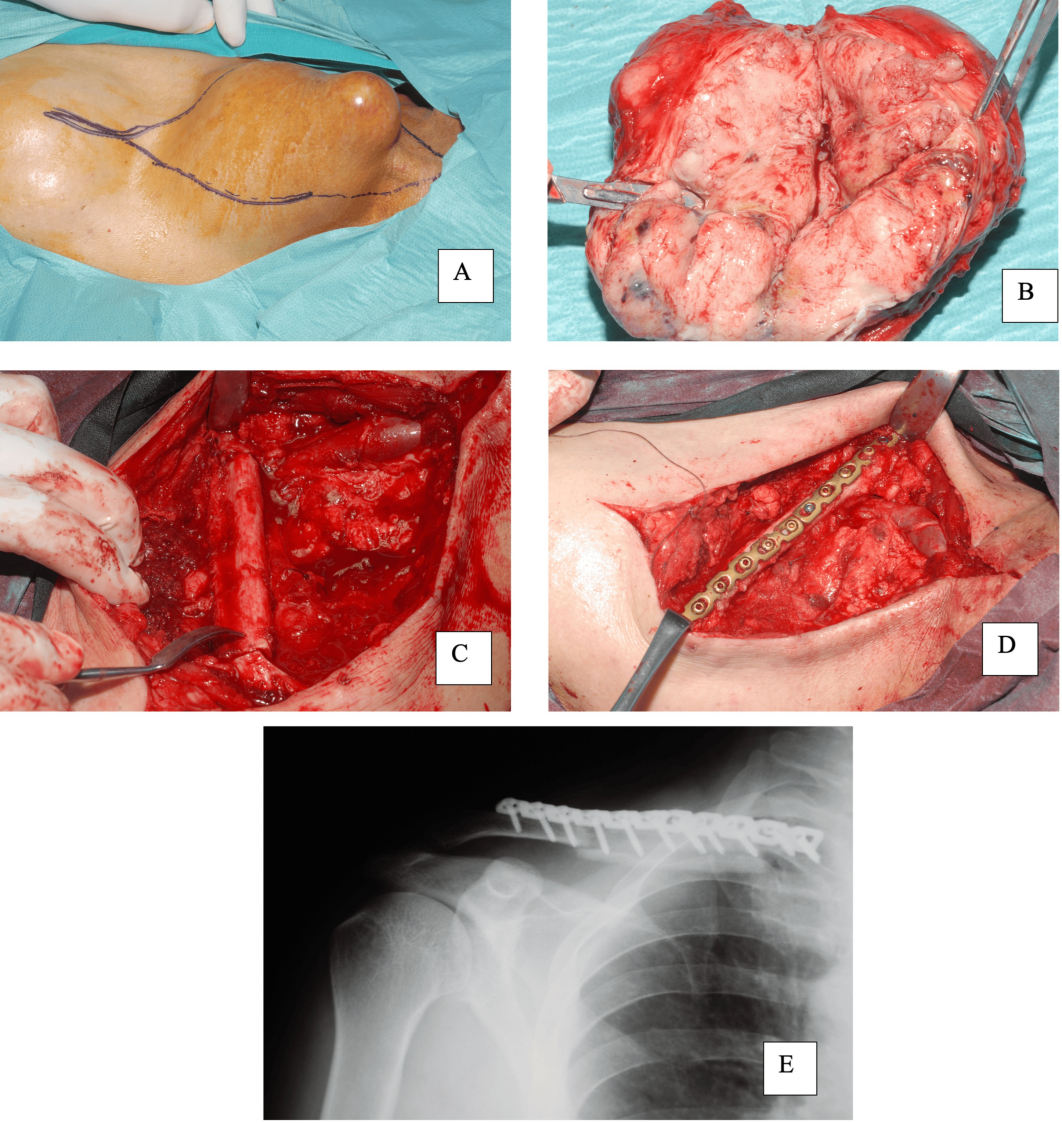
Pre- intra and post-operative images A: Preoperative skin marking outlining the planned incision for en bloc clavicular resection. B: Gross specimen showing the gray-white excised tumor without any surrounding invasion of the local tissues. C, D: Reconstruction and fixation following resection of the tumor. E: Postoperative X-ray.

A fine-needle bone biopsy was performed, yielding a preliminary diagnosis of grade II chondrosarcoma. Staging studies revealed no evidence of metastatic disease, and a decision was made to proceed with radical surgical resection. The tumor and the involved segment of the clavicular diaphysis were excised en bloc with wide margins through uninvolved tissue. The resected specimen measured 13×12×7 cm and included an approximately 8 cm segment of the clavicular diaphysis. Reconstruction of the resultant defect was achieved using a non-vascularized fibular autograft stabilized with two 3.5 mm plates (Figure 5). The plates were applied in series due to the length of the segmental defect, allowing adequate fixation on either side of the fibular graft. Reconstruction plates were selected for their ability to be contoured in two planes, facilitating adaptation to the clavicle's complex curvature and providing a stable construct across the graft-host interfaces.

Final histopathological assessment of the surgical specimen revealed a neoplastic proliferation composed of medium-sized, relatively amorphous cells with weakly eosinophilic to focally clear cytoplasm. Nuclei were sparsely chromatic, subround, and exhibited indistinct nucleoli, with only rare mitotic figures. The neoplasm was partially surrounded by a fibrous pseudocapsule that was not infiltrated. All surgical margins, including the adjacent skin segment, were free of tumor. Immunohistochemically, the tumor cells showed strong expression of vimentin, CD10, neuron-specific enolase (NSE), calponin, desmin (focal), synaptophysin, CD56, S-100 (dot-like), keratin AE1/AE3, keratin 7, actin, and Ki-67 (~3%). They were negative for keratins 5/6, 8/18, and 19, epithelial membrane antigen (EMA), B-cell lymphoma 2 (BCL-2), human melanoma black 45​​​​​​​(HMB-45), estrogen and progesterone receptors, CD31, CD34, CD117, CD68, CD99, smooth muscle actin​​​​​​​ (SMA), CD45, myogenic differentiation 1​​​​​​​ (MyoD1), myogenin, glial fibrillary acidic protein​​​​​​​ (GFAP), CD57, CD23, CD138, terminal deoxynucleotidyl transferase​​​​​​​ (TdT), thyroid transcription factor-1​​​​​​​ (TTF-1), tumor protein p63​​​​​​​ (p63), myeloperoxidase​​​​​​​ (MPO), and carcinoembryonic antigen​​​​​​​ (CEA). Taken together, the morphological and immunophenotypic profiles supported the diagnosis of clear cell myoepithelial carcinoma of bone.

The patient's postoperative course was uneventful. At six months postoperatively, he achieved active shoulder forward flexion to approximately 160°, abduction to 150°, and near-complete internal and external rotation, without neurovascular deficits or wound complications. Radiographs obtained at follow-up demonstrated stable fixation and satisfactory incorporation of the fibular graft. At six months, the patient remained pain-free, with progressive functional improvement and no evidence of local recurrence or metastatic disease on surveillance imaging.

## Discussion

Primary malignant tumors of the clavicle account for fewer than 1% of all bone neoplasms, making them diagnostically challenging and often unfamiliar to clinicians [9]. Their rarity results in limited cumulative experience regarding optimal evaluation, surgical planning, and long-term surveillance. Consequently, the diagnostic approach to clavicular lesions must be systematic and include a broad differential diagnosis, taking into consideration the tumor's origin (osseous vs. soft tissue), biological aggressiveness, size, and the proximity of vital anatomical structures. Bone myoepithelial carcinomas must be distinguished from other benign and malignant bone and cartilage-forming surface tumors, including periosteal chondroma and chondrosarcoma, juxtacortical chondromyxoid fibroma, and periosteal and paraosteal osteosarcoma. In the present case, the lobulated morphology, presence of internal calcified foci consistent with a chondroid matrix, and pressure remodeling of the underlying cortex favored an initial diagnosis of chondrosarcoma. This impression was further reinforced by the initial needle biopsy, which suggested grade II chondrosarcoma, but ultimately differed from the final histopathologic diagnosis. Such discrepancies highlight the limitations of biopsy in uncommon and heterogeneous bone lesions, where limited sampling may not capture the full histologic spectrum of the tumor; other options include core needle biopsy and open biopsy, which may provide more representative tissue for diagnosis [2].

Furthermore, immunohistochemistry plays a pivotal role in the diagnosis of myoepithelial carcinoma, particularly in extra-salivary and osseous locations where morphologic overlap with other entities is common. Expression of both epithelial and myoepithelial markers, including S-100 protein, calponin, actin, and, variably, desmin, supports myoepithelial differentiation. In the present case, the combined expression of keratin AE1/AE3, S-100 (dot-like), calponin, and actin was critical in establishing the diagnosis. The absence of lineage-specific markers, including EMA, CD34, CD31, myogenic markers (MyoD1, myogenin), melanocytic markers (HMB-45), and lymphoid markers (CD45), helped exclude alternative diagnoses such as sarcomas and hematologic malignancies. Given the scarcity of published cases, each clavicular malignancy provides valuable data that can assist in refining diagnostic criteria, operative strategies, and postoperative management.

Bone myoepithelial carcinoma is itself a sparsely reported entity with a wide age distribution and no significant sex predilection [2]. Pediatric variants tend to behave more aggressively and demonstrate higher rates of malignancy compared to adult tumors. Overall, malignant myoepithelial neoplasms exhibit a propensity for local recurrence and, in some cases, distant metastasis, underscoring the importance of complete surgical excision as the primary prognostic determinant [10]. Only a limited number of primary osseous myoepithelial tumors have been reported in the literature. Franchi et al. [11] described three cases of primary juxtacortical myoepithelioma/mixed tumor of bone, all arising in the femur of adolescent and young adult patients, presenting as surface-based, lobulated lesions with chondromyxoid or chondroid matrix and cortical involvement. Similar to the present case, these tumors demonstrated diagnostic difficulty on imaging and required surgical excision for definitive diagnosis. In contrast to our patient, the previously reported cases occurred in younger individuals, involved the femur, and were classified as myoepithelioma or mixed tumor rather than clear cell myoepithelial carcinoma. One case showed local recurrence following marginal excision, underscoring the importance of wide resection. Our case further expands the spectrum of reported osseous myoepithelial neoplasms by demonstrating clavicular involvement, a prolonged indolent clinical course, and favorable outcomes following wide oncologic resection.

From a surgical standpoint, the unique regional anatomy, lying immediately above the brachial plexus, subclavian vessels, and apex of the lung, necessitates meticulous preoperative planning [12]. Achieving wide, margin-negative excision remains the mainstay of treatment, but surgeons must simultaneously anticipate the functional implications of the resulting defect and the potential need for structural restoration. However, such restoration is not mandatory in every case. In selected patients with limited segmental resections, preserved soft-tissue envelope, and low functional demands, claviculectomy without reconstruction may yield acceptable functional outcomes. By contrast, when large segmental defects are created, particularly in active patients, reconstruction may be warranted to restore shoulder girdle biomechanics, maintain clavicular contour, and protect adjacent neurovascular structures. In such cases, the fibular autograft remains one of the most widely described options, offering reliable biological incorporation and adequate strength capable of bridging extensive defects [13,14]. Alternative strategies include plate fixation without grafting and allograft bone utilization; however, these approaches may provide limited structural support and less favorable biological potential for incorporation in long defects. Fixation with one or two compression plates is commonly reported, although alternative methods have been used depending on surgeon preference and defect size [15]. In the present case, reconstruction with a fibular autograft provided both mechanical stability and protection of adjacent neurovascular structures, aligning with established principles for the management of extensive clavicular defects following oncologic resection. 

## Conclusions

Clear cell myoepithelial carcinoma of bone is extremely rare, and clavicular involvement is exceptional, often complicating diagnosis and management. Definitive diagnosis requires careful histopathologic and immunohistochemical assessment, while wide, margin-negative excision remains the cornerstone of treatment. Reconstruction should be tailored to the defect and regional anatomy to preserve function and protect vital structures. This case underscores the importance of considering rare malignancies in atypical clavicular lesions and highlights the effectiveness of radical resection with appropriate reconstruction. In practice, clavicular lesions with indeterminate imaging features should prompt heightened diagnostic suspicion and consideration of more representative tissue sampling when initial biopsy results are inconclusive.
